# Prostac: A New Composite Score With Potential Predictive Value in Prostate Cancer

**DOI:** 10.3389/fonc.2021.644665

**Published:** 2021-03-16

**Authors:** E. O. Asante-Asamani, Gargi Pal, Leslie Liu, Olorunseun O. Ogunwobi

**Affiliations:** ^1^ Department of Mathematics, Clarkson University, Potsdam, NY, United States; ^2^ Department of Biological Sciences, Hunter College of The City University of New York, New York, NY, United States; ^3^ Value based payment unit, Fidelis Care/Centene, Long Island City, NY, United States; ^4^ Department of Medicine, Weill Cornell Medicine, New York, NY, United States

**Keywords:** composite score, PVT1 exons, biomarkers, prostate cancer, mathematical oncology, support vector machines

## Abstract

Prostate cancer (PCa) is the most commonly diagnosed solid organ cancer in men worldwide. Current diagnosis of PCa includes use of initial prostate specific antigen assay which has a high false positive rate, low specificity, and low sensitivity. The side effects of unnecessary prostate biopsies that healthy men are subjected to, often result in unintended health complications. New PCa biomarkers are being discovered to address this unmet need. Here, we report on the creation of a composite score (Prostac) based on three recently discovered PCa biomarkers, Plasmacytoma Variant Translocation 1 (PVT1) exons 4A, 4B, and 9. Statistical analysis of copy numbers derived from a real-time quantitative polymerase chain (qPCR) reaction - based assay, showed these PCa biomarkers to be linearly separable and significantly over expressed in PCa epithelial cells. We train a supervised learning algorithm using support vector machines to generate a classification hyperplane from which a user-friendly composite score is developed. Cross validation of Prostac using data from prostate epithelial cells (RWPE1) and PCa cells (MDA PCa 2b) accurately classified 100% of PCa cells. Creation of the Prostac score lays the groundwork for clinical trial of its use in PCa diagnosis.

## Introduction

Prostate cancer (PCa) is the leading form of cancer in men in the United States, resulting in the second largest number of deaths by cancer in men (1, 2). In 2021, 248,530,930 men are projected to be diagnosed with prostate cancer, 34,130 of whom are expected to die from the disease (1). The disease disproportionately affects men of African ancestry (moAA) who are not just more likely get the disease but also more likely to die from it (1). An effective way to curb cancer mortality is to detect it early and initiate treatment, yet one of the most widely used screening tests for prostate cancer, prostate specific antigen test (PSA), has a high false positive rate (3–5). The unintended health complications from the side effects of unnecessary prostate biopsies resulting from the high false positive rate of PSA, has spurred a growing interest in new biomarkers. The preferred biomarker is one acquired through noninvasive methods that can more effectively isolate indolent from aggressive forms of PCa, so that commensurate management and treatment protocols can be applied (6, 7).

Plasmacytoma variant translocation 1 (PVT1) is one of the transcribed long non-coding RNAs located on the 8q24 human chromosomal region (8, 9). It has been shown to play an important role in colorectal cancer (10), gastric cancer (11), lung cancer (12), and prostate cancer (13). Recently, 3 of its 12 exons, such as, PVT1 exons 4A, 4B, and 9 were shown to be significantly over expressed in moAA with PCa (14–16). In particular, PVT1 exon 9 was shown to be significantly overexpressed in moAA with aggressive PCa (14). The promise of these potential biomarkers to improve the clinical diagnosis of PCa, among this most vulnerable population group, was subsequently elevated by the development of a non-invasive copy number-based quantification assay for detecting PVT1-derived transcripts from prostate tissues, serum, and urine samples (17). Whereas expression levels of PVT1 exons 4A, 4B, and 9 are promising biomarkers in their own right, it is reasonable to expect that a single numeric score that combines information from all two or more transcripts would be a more robust and easier to use diagnostic tool for clinicians.

The development of such an aggregate score and an evaluation of its efficacy is often done with machine learning techniques that have in recent years revolutionized healthcare (18). These techniques use data to learn parameters in a statistical model that can capture relationships between factors (features) that influence a particular clinical outcome (19). They have been used to improve early detection and diagnosis, treatment as well as outcome prediction and prognosis evaluation of several diseases including cancer (7, 20, 21), nervous system disease (22) and cardiovascular disease (23). The most common machine learning techniques used in the medical literature are support vector machines, neural networks and logistic regression, with support vector machines being the most widely used (18).

Support vector machines (SVM) are used to classify observations into a finite number of classes by separating the feature space or some transformation of it with a hyperplane (19). When a hyperplane exists that can perfectly separate the feature space without transformation, the resulting classifier is called a maximal margin classifier. Such a perfect classification of training data can sometimes lead to sensitivity of the separating hyperplane to changes in the training data and result in a poor predictive performance on test data. An alternative to the maximal margin classifier is the support vector classifier which allows some degree of misclassification of the training data in order to improve the robustness of the model. The support vector classifier is also used when all, but a few features are linearly separable. In cases where most of the data cannot be separated by a hyperplane, a nonlinear decision boundary can be obtained by an appropriate transformation of the feature space. The resulting classifiers are support vector machines, a description often used for all three methods. SVM have been successfully used to diagnose cancer (24) as well as neurological disorders from imaging biomarkers (25, 26).

In this study, we apply support vector classifiers to develop a composite score for detecting PCa using expression levels of PVT1 exons 4A, 4B, and 9 derived from a non-tumorigenic prostate epithelial cell from a Caucasian male and a tumorigenic prostate cancer cell line derived from a moAA. The Prostac score correctly predicted the incidence of PCa in 100% of observations we tested, a result that lays the groundwork for clinical trial of its use in PCa diagnosis in larger more heterogeneous populations.

## Materials and Methods

### Cell Culture and Cell Culture Reagents

Transcription data on the non-coding RNA PVT1 were obtained from two cell lines. First, a non-tumorigenic prostate epithelial cell derived from a Caucasian male (RWPE-1). Second, a tumorigenic prostate cancer cell line derived from a moAA (MDA PCa 2b). These two cell lines were selected after an initial qPCR screening of different types of prostate epithelial cell lines, non-tumorigenic epithelial, mildly tumorigenic, and metastatic prostate cancer derived from African as well as Caucasian populations. They showed the greatest difference in expression levels of PVT1 exons 4A, 4B and 9. RWPE-1 cells were cultured in keratinocyte-serum free medium (SFM) supplemented with 0.05mg/ml bovine pituitary extract (BPE), 5ng/ml epidermal growth factor (EGF) and 1% penicillin/streptomycin. MDA PCa 2b cells were cultured in F-12K medium supplemented with 20% fetal bovine serum (FBS), 25 ng/ml cholera toxin, 10ng/ml mouse epidermal growth factor, 0.005mM phosphoethanolamine, 100pg/ml hydrocortisone, 45 nM selenious acid and 0.005 mg/ml bovine insulin.

### RNA Extraction

Total RNA was extracted from all cell lines at 75% confluency in a 60x15 mm tissue culture dish using RNeasy Mini Kit (Qiagen, Germany, cat #74104). The RNA was quantified using a Nanodrop 1000 spectrophotometer (NanoDrop, Madison, WI, USA). 500 ng of RNA was reverse transcribed into cDNA using QuantiTect Reverse Transcription Kit with random hexamers (Qiagen, Germany, cat #205311).

### Copy Number-Based Quantification Assay

The quantitative PCR assay was performed on an ABI 7500 platform (Applied Biosystems instruments Grand Island, NY, USA). Primers for PVT1 exons 4A, 4B, and 9 were designed using Primer3Plus. The assay was performed as previously described (17). Concentration of PCR products were measured spectrophotometrically using NanoDrop^®^ ND100 (Thermo Scientific NanoDrop Products, Wilmington, Delaware) in nanograms per microliter and converted to copies per microliter using the formula

$$y\frac{copies}{\mu l}=\left(\frac{\left[x\frac{ng}{\mu l}\times 10^{-9}\right]}{\left[pcDNA\;vector\text{ and }273|301|130\;DNA\;bps\;\times 660\right]}\right)\times 6.022\times 10^{23}$$

### Construction of Standard Curve and Data Collection

Serial dilutions of the PCR products for each exon were prepared within the range 10^1^ – 10^10^ copies/µl. A linear regression line was fit to the Ct values and the log_10_ copies/µl. This regression line was used to estimate the concentration of transcripts (copies/µl) directly from the measured Ct values. For each cell type, 10 sets of qPCRs were performed for each of the three PVT1 exons. The Ct values for each set was calculated as the average of four repeated measurements. A standard curve was generated for each set of qPCRs and used to calculate the copies/µl of the transcripts. In all there were 20 sample copies/µl in each of PVT1 exons 4A, 4B, and 9. Samples from RWPE-1 were identified with -1 and 1 for samples from MDA PCa 2b.

### Data Analysis

Statistical analysis was carried out using RStudio software (Version 1.2.5003). Pearson correlation coefficient was used to access the level of correlation between each pair of biomarkers. The *svm()* function from the e1071 library in R was used to fit support vector classifiers. 10-fold cross validation was performed to estimate the test error rate of all models constructed. Variability of predictive performance was evaluated by repeated 10-fold cross validations on all 20 samples. The best model was chosen to give the least variability with minimal error rate. The equation of the best fitted hyperplane was used as the composite score. Positive model outputs were predictive of PCa while negative outputs predicted healthy prostate epithelial cells.

Our data analysis was performed using 20 samples. While this sample size might be considered small for most machine learning tasks, we do not believe collecting more data would improve the current model for the following reasons. First, the concentration of biomarkers is obtained from populations of two cell lines, both of which are genetically identical within their respective populations. As such we do not expect significant variation in the measured concentration of biomarkers within each population. Secondly, we constructed our decision boundary using support vector classifiers which maximize the distance between the separating hyperplane and the nearest observation while allowing for some misclassification in the training data. This helps to reduce the sensitivity of the model to small variations in the training data (19). Thirdly, to reduce training bias in our model, we estimated the prediction error rates by performing 10 sets of 10 -fold cross validation on all 20 samples.

### Support Vector Machines

Consider the data $({\bar{x}}_{i},y_{i}),\;i=1,2\cdots n$ with input features ${\bar{x}}_{i}=(x_{1i},x_{2i},x_{3i},\cdots x_{mi})$ and a binary outcome *y_i_* taking on the values -1 or 1. In our application the features represent the measured copies/µl of each biomarker (PVTI exons 4A, 4B, and 9), with the presence or absence of PCa in each sample as our outcome *y_i_*. If there exists a hyperplane $f_{\beta }(\bar{s})=0$, $f_{\beta }(\bar{s})=\beta_{0}+\beta_{1}s_{1}+\beta_{2}s_{2}+\cdots \beta_{m}s_{m}$ with $\beta =(\beta_{0},\beta_{1},\cdots ,\beta_{m})$ a real vector, for which $f_{\beta }({\bar{x}}_{i})>0\;if\;y_{i}=1$ and $f_{\beta }({\bar{x}}_{i})<0\;if\;y_{i}=-1$ for all observations then the data is said to be linearly separable and $f_{\beta }(\bar{s})$ is called the *separating hyperplane*. If the hyperplane is chosen so that $\Sigma_{k=0}^{m}\;\beta_{k}^{2}=1$ then the perpendicular distance between each observation ${\bar{x}}_{i}$ and the hyperplane is given by $y_{i}\cdot f_{\beta }({\bar{x}}_{i})=M_{i},M_{i}>0$ (27). Let $M=\underset{i}{\min}M_{i}$, the minimum separating distance over all observations, then the hyperplane for which M is the largest, over all possible parameters *β*, is referred to as the *maximal margin classifier*, in which case all observations will be at least M distance away from the separating hyperplane. The distance M is referred to as the *margin* and the observations which lie on this margin are called *support vectors.* The maximal margin classifier achieves two things. Every training observation will be on the right side of the separating hyperplane as well as outside of the margin. The disadvantage of this approach is that a change in a single observation can significantly alter the hyperplane. The maximal margin classifier is therefore likely to underperform when applied to test data.

If the data is not linearly separable then we expect that for any hyperplane constructed there will be a subset of observations on the wrong side of the hyperplane $\left(f_{\beta }({\bar{x}}_{i})<0\;for\;y_{i}=1\;and/or\;f_{\beta }({\bar{x}}_{i})>0\;for\;y_{i}=-1\right)$, without a separating margin (*M* = 0). The best hyperplane in this case, will be one which minimizes the number of misclassifications (observations on the wrong side of the margin) while keeping observations as far away from the hyperplane as possible. This is the *support vector classifier.* This method is also applied even when the data are linearly separable in an effort to increase the robustness of the classifier to unseen test data. A broader class of methods achieves a reasonable hyperplane by first transforming the feature space ${\bar{x}}_{i}\to H({\bar{x}}_{i})$. The resulting classifier is called a *support vector machine*. In this work, we allowed for the possibility of a small number of misclassifications to occur in our training data and so constructed a classification hyperplane using support vector classifiers.

## Results

We sought to construct and validate a composite score for detection of PCa using the transcription levels of PVT1 exons 4A, 4B, and 9. The concentration (copies/µl) was measured using the copy number-based quantification assay from 10 prostate epithelial cell samples (RWPE-1) and 10 PCa cell samples (MDA PCa 2b). See **S1 Table** for complete data.

### Elevated Copy Numbers of PVT1 Exons 4A, 4B, and 9 in Prostate Cancer Cells

**Figure 1:**
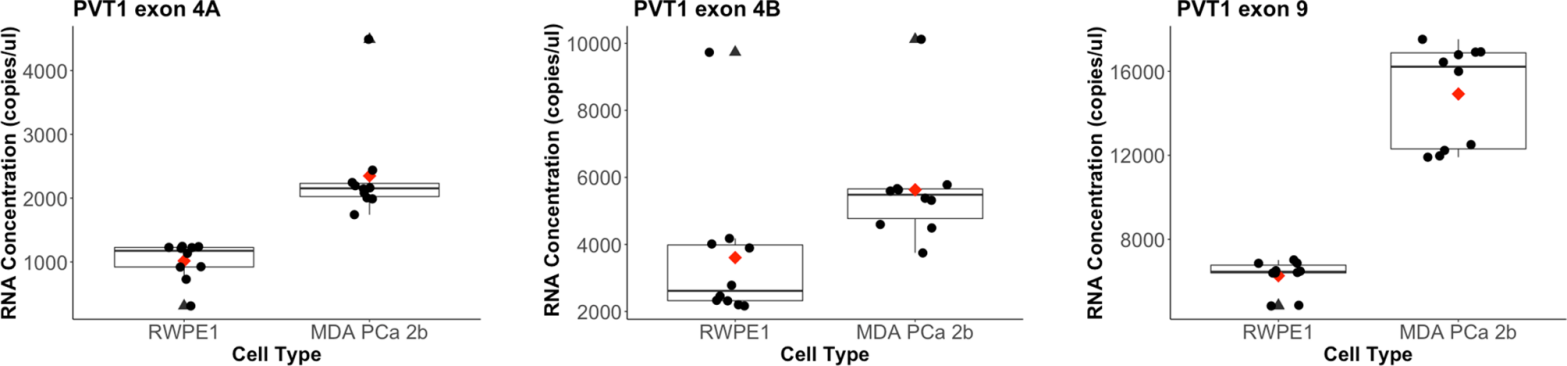
Distribution of biomarkers measured from the copy number-based quantification assay for a prostate epithelial cell (RWPE-1) and a PCa cell line (MDA PCa 2b). PVT1 exon 4A levels in RWPE-1 (mean: 1017, standard deviation: 290) and in MDA PCa 2b (mean: 2349.2, standard deviation: 773.68). Outlier for MDA PCa 2b is at 4489 copies/µl. PVT1 exon 4B levels in RWPE-1 (mean: 3607, standard deviation: 2179) and in MDA PCa 2b (mean: 5630.5, standard deviation: 1706.936). Outlier for RWPE-1 is 9733 copies/µl and 10117 copies/µl for MDA PCa 2b). PVT1 exon 9 levels in RWPE-1 (mean: 6263, standard deviation: 740) and in MDA PCa 2b (mean: 14921.19, standard deviation: 2414.469). Mean values are shown as red diamonds within each category in the boxplot. Outliers are shown as black triangles. Data is displayed as black dots.

The copies/µl of all three biomarkers were significantly higher in MDA PCa 2b cells than in RWPE1 cells, as confirmed by the Mann-Whitney test (*α* = 0.05). PVT1 exon 4A in the PCa samples was 2.3 times higher than in the RWPE1 cell (p value =1.418e-06), PVT1 exon 4B was 1.5 times higher (p value = 0.00052) while PVT1 exon 9 was 2.4 times higher (p value = 5.413e-06). The distribution of the data is shown in **Figure 1**. We evaluated the performance of each of these biomarkers in diagnosing PCa on the training data (15 observations). PVT1 exon 4A predicted PCa cases with sensitivity of 1 and specificity of 1 (cutoff, 1618 copies/µl). Predictions with PVT1 exon 4B were not as accurate with specificity of 0.5 and sensitivity of 1 (cutoff, 3549 copies/µl). Prediction accuracy for PVT1 exon 9 was identical to PVT1 exon 4A (cutoff, 9463 copies/µl).

### Copy Numbers of PVT1 Exons 4A and 9 Have a Strong Positive Correlation

In order to access any dependences between the biomarkers which could render some of them redundant, we examined their pairwise correlation and found transcripts from PVT1 exons 4A and 9 to be positively correlated with Pearson correlation coefficient 0.66 (p value < 0.01). PVT1 exons 4A and 4B showed a statistically significant weak positive correlation with coefficient 0.47 (p value < 0.05). No significant correlation was observed for PVT1 exons 4B and 9 (**S1 Fig 1**).

### Composite Score Using Copy Numbers of PVT1 Exons 4A, 4B, and 9 Holds Promise

**Figure 2:**
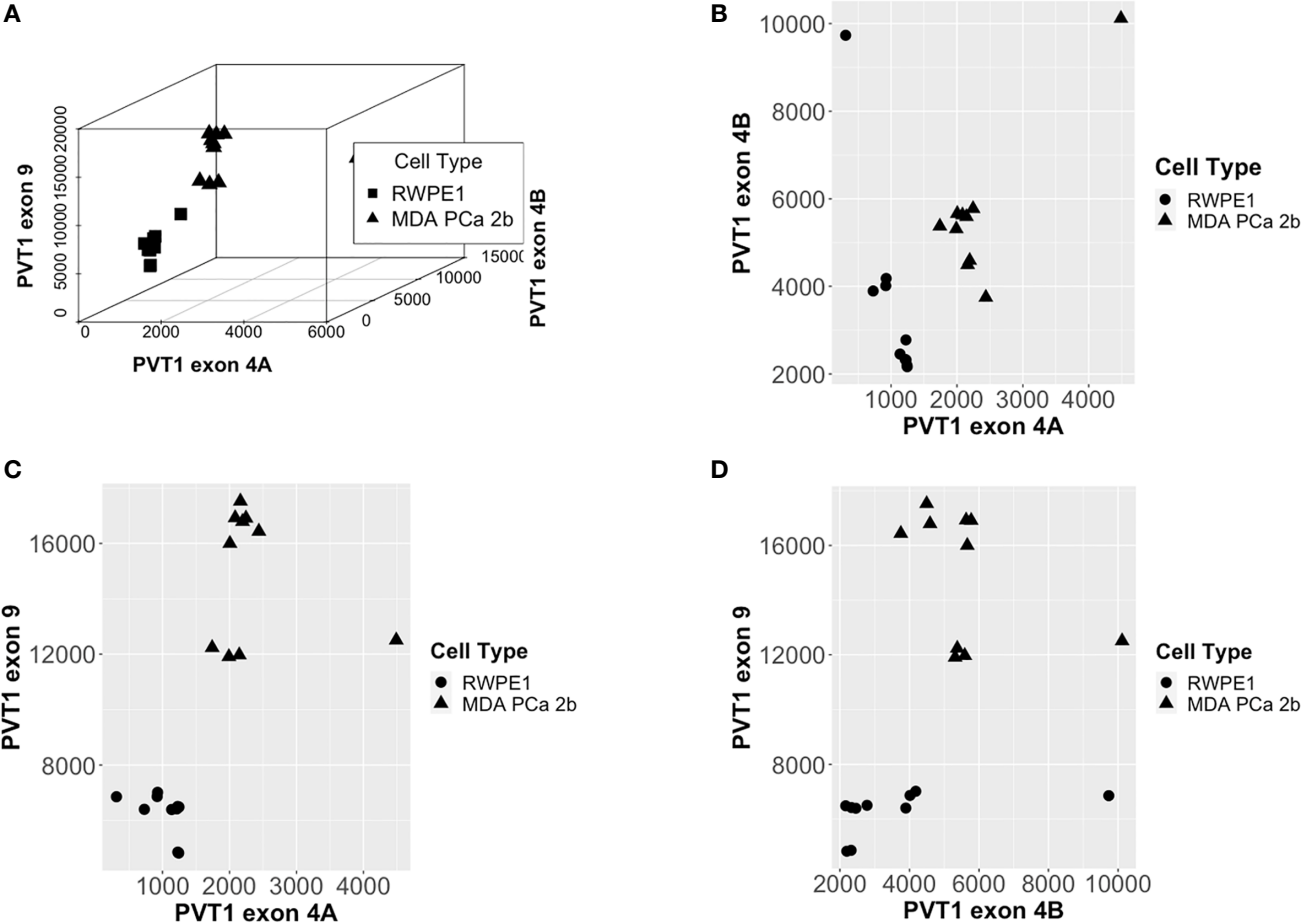
PCa Biomarkers are linearly separable by cell type. **(A)** Copies/µl of PVT1 exons 4A, 4B, and 9. **(B)** Copies/µl of PVT1 exon 4A and 4B. **(C)** Copies/µl of PVT1 exons 4A and 9. **(D)** Copies/µl of PVT1 exons 4B and 9.

**Figure 3:**
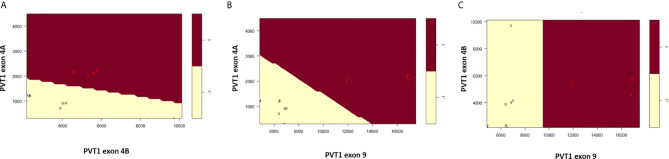
Linear decision boundary generated from support vector machines perfectly classifies RWPE1 (-1) and MDA PCa 2b (1). **(A)** Classification based on PVT1 exons 4A and 4B. **(B)** Classification based on PVT1 exons 4A and 9. **(C)** Classification based on PVT1 exons 4B and 9. Data is shown as circles with support vectors as x’s.

Scatter plots of the copies/µl of biomarkers revealed the existence of a linear decision boundary separating the MDA PCa 2b cases from the RWPE1 ones **Figure 2**. Consequently, we trained four different support vector classifiers constructed by using all three biomarkers and all possible pairs of biomarkers. The models are summarized in **Table 1**. All the models returned a training error rate of 0. The decision boundaries for model 1, model 2 and model 3 generated from a training set of 15 observations and a misclassification cost of 1 are shown in **Figure 3**. Since the training error rate is based on a particular random sample of our observations and may not necessarily reflect the performance of the model on previously unseen data, we sought to narrow down the potential models by investigating the variability in the validation error rates using cross validation. We performed 10 sets of 10-fold cross validation on all 20 observations. All the models showed remarkable robustness with Model 2 and Model 4 yielding zero error rates across all random samples (**Table 2**).

**Table 1:** Composite marker models used for classification.

Model	Biomarkers used	Model form
Model 1	PVT1 exon 4A and PVT1 exon 4B	*PCa*~*c*_1_ + *c*_2_ *Exon* 4*A* + *c*_3_ *Exon*4*B*
Model 2	PVT1 exon 4A and PVT1 exon 9	*PCa*~*c*_1_ + *c*_2_ *Exon* 4*A* + *c*_3_ *Exon* 9
Model 3	PVT1 exon 4B and PVT1 exon 9	*PCa*~*c*_1_ + *c*_2_ *Exon* 4*B* + *c*_3_ *Exon* 9
Model 4	PVT1 exon 4A, PVT1 exon 4B, PVT1 exon 9	*PCa*~*c*_1_ + *c*_2_ *Exon* 4*A* + *c*_3_ *Exon*4*B* + *c*_4_ *Exon* 9

**Table 2:** Variability in 10-fold cross validation error rate with tunning parameter of 1.

Model/Trial	1	2	3	4	5	6	7	8	9	10
**1**	0.05	0.05	0.05	0.05	0.05	0.05	0.05	0.05	0.05	0.05
**2**	0	0	0	0	0	0	0	0	0	0
**3**	0.05	0.05	0.05	0.05	0.05	0.05	0.05	0.05	0.05	0.05
**4**	0	0	0	0	0	0	0	0	0	0

An important parameter in fitting support vector classifiers is the cost of misclassifications or the tuning parameter. Higher values for the tuning parameter allow for more misclassifications in the training data but often leads to a better classification of test data (19). We were also concerned about the number of support vectors used for constructing the separating hyperplane. The greater the number of support vectors the more sensitive the decision boundary is to changes in the training data. Such models tend to overfit the training data and consequently do not classify unseen data well (27). Our goal here was to choose an aggregate model with the smallest possible tuning parameter which yields a minimal test error rate with as few support vectors as possible. Again, we performed a 10-fold cross validation for Model 2 and Model 4 but this time, we varied the tuning parameter to range from 0.01-100. Both Model 2 and Model 4 had zero error rate for tuning parameters at or above 0.1, but had a large number of support vectors for tunning parameter values at or below 0.1 **Table 3**. At a tuning parameter value of 1 both Model 2 and Model 4 had relatively low number of support vectors, making them both ideal.

**Table 3:** Error rate of competing model across different levels of model flexibility.

Model 2						
Tunning Parameter	0.001	0.01	0.1	1	5	10	100
Error rate	0.25	0.25	0	0	0	0	0
Support vectors	18	18	10	4	2	2	2
**Model 4**								
Tuning Parameter	0.001	0.01	0.1	1	5	10	100
Error rate	0.80	0.75	0	0	0	0	0
Support Vectors	18	18	10	3	3	3	3

The predictive performance of these two competing models is however similar to the performance of PVT1 exons 4A and 9 as individual markers, which both had a cross validation error rate of zero. Since model 2 combines information from PVT1 exons 4A and 9 but does not achieve a better performance, the individual markers should be preferred. The strong positive correlation between PVT1 exons 4A and 9 suggests that either model should suffice as a single predictive marker of PCa. Our current data however do not distinguish between the performance of the single markers and the 3-marker score. We expect the extra flexibility provided by the addition of PVT1 exon 4B to increase the robustness of the model for clinical settings. The proposed Prostac model for predicting the presence of PCa is thus model 4 which is given by the expression,

$$PCaDiag=-3.87+9.05\times 10^{-5}\;PVT1exon4A+2.10\times 10^{-5}\;PVT1\;exon4B+3.84\times 10^{-4}PVT1\;exon9$$

where observations with PCaDiag > 0 are classified as cancerous and noncancerous if PCaDiag<0.

## Discussion

We have developed the Prostac score of three non-protein coding RNAs transcribed from PVT1 exons 4A, 4B, and 9 of the PVT1 gene locus. The expression levels of all three exons were significantly higher in MDA PCa 2b cells than in RWPE1 cells (**Figure 1**). This is consistent with our previous observation of overexpression of these non-protein coding RNAs in PCa tissues (14, 16). We found the concentration of PVT1 exons 4A and 9 to be positively correlated across both MDA PCa 2b and RWPE1 cells. In recent studies, the overexpression of PVT1 exons 4A and 9 have both been associated with increased epithelial cell migration and increased proliferation (15, 16). PVT1 exon 9 was also found to induce the formation of malignant tumors in mice, a phenotype that strongly suggests an association with aggressive forms of PCa (15). The strong positive correlation between PVT1 exons 4A and 9 together with our observation of both having a mean concentration (copies/µl) about 2.4 times higher in PCa, suggests that PVT1 exon 4A may also be indicative of an aggressive form of PCa. It not surprising therefore that we obtained near perfect predictability from composite scores involving these two exons (Model 2, PVT1 exon 4A with cut of 1618 copies/µl, and PVT1 exon 9 with cutoff 9463 copies/µl). Both biomarkers had a sensitivity and specificity of 1, suggesting that either of them would be sufficient to perfectly identify PCa cells. However, since this is a laboratory study with two cell lines, we have chosen to include both of these biomarkers in our composite score in anticipation of increased robustness in predictability when applied on a population level.

On the other hand, the mean concentration (copies/µl) of PVT1 exon 4B in PCa cells examined in this study was only 1.5 times higher than in RWPE1 cells and was a relatively weak discriminator of PCa from normal epithelial cells. It would have made sense to remove it from our 3-marker composite score since the 2-marker score with just PVT1 exon 9 and PVT1 exon 4A had an error rate similar to the 3-marker score. However, we recently reported that PVT1 exon 4B is significantly over expressed in prostate tumors with Gleason score ≥8 as compared to those with Gleason score ≤7 (16), suggesting that PVT1 exon 4B expression may be well suited for distinguishing between indolent and aggressive PCa. In the absence of data to fully test our assertion, we have chosen to leave PVT1 exon 4B in our composite score in anticipation of improving our ability to distinguish between indolent and aggressive PCa.

Our results show that the proposed Prostac score can perfectly detect the occurrence of PCa in MDA PCA 2b cell lines with a strong potential to accurately detect PCa in populations of males of African ancestry. All of the biomarkers in our score have been shown to increase proliferative and migratory capacity when overexpressed in the nontumorigenic prostate epithelial cell line (RWPE1) obtained from a Caucasian male (15, 16). In particular, PVT1 exon 9 has been shown to increase proliferation by upregulating the expression of proliferating cell nuclear antigen (PCNA) (15). The same mechanism was shown to be at play in the tumorigenic prostate cancer cell line (MDA PCa 2b), which significantly reduced its PCNA expression when PVT1 exon 9 expression was silenced (15). Since increased proliferation is characteristic of cancer cells and has been shown to be regulated by PVT1 exon 9 which is positively correlated with PVT1 exon 4A, we suspect that our score may be predictive of PCa in other races. In the future, we intend to fully validate the clinically efficacy of the Prostac score in detecting PCa and investigate its potential in distinguishing between aggressive and indolent forms of PCa, by repeating our analysis with data from larger, more heterogeneous populations. Clinical use of the final score could involve a non-invasive collection of a urine or serum sample, after which a nucleic acid amplification test (qRT-PCR) using PVT1 primers will be used to quantify the concentration (in copies/µl) of biomarkers present. The concentrations can then be fed into the mathematical formula for diagnosis.

While the Prostac composite score may have promise for potential utility in predicting positive PCa prostate biopsy and other clinical applications in PCa, we acknowledge that there are significant limitations of our current report. Most notably, our results analyzed and discussed in this article were based on direct analysis of data from human prostate cell lines, and not human prostate tissues, urine, or serum. Therefore, it is possible that the data presented may not apply to human prostate cancer. To definitively address this important limitation, analysis of data from human prostate tissues, urine, or serum must be performed.

We have laid out a framework within which support vector machines can be used to generate a composite score for prostate cancer. While this methodology may appear over complicated for the task at hand, its versatility permits an easy extension of our analysis to much larger more heterogeneous populations. Here, our data was linearly separable, so we applied support vector classifiers to generate a separating hyperplane. Future data exhibiting a more nonlinear structure will be analyzed using nonlinear kernels in support vector machines.

## Data Availability Statement 

The original contributions presented in the study are included in the article/**Supplementary Material**. Further inquiries can be directed to the corresponding author.

## Author Contributions

Conceptualization: OO. Funding acquisition: OO. Formal Analysis: EA-A, LL. Investigation: OO, GP. Writing – Original draft: EA-A. Writing – Review and Editing: OO, GP. All authors contributed to the article and approved the submitted version.

## Funding

OO is supported by the National Cancer Institute grant number U54CA221704.

## Conflict of Interest

LL was employed by Fidelis Care. OO is Co-Founder of NucleoBio, Inc, a City University of New York start-up biotechnology company.

The remaining authors declare that the research was conducted in the absence of any commercial or financial relationships that could be construed as a potential conflict of interest.

## Supplementary Material

Supplementary Figure 1 Correlation tests between exons. Pearson correlation matrix for PVT1 exons 4A, 4B, and 9. Mapping from p-values to symbols is as follows: ***(0-0.001), **(0.001-0.01), *(0.01-0.05), (0.05-0.1). Histograms show the distribution of the data.

Supplementary Table 1 Measured Copies/µl of exon 4A, 4B, and 9 in copies/µl.
