# Supplementary figures and images for: Prostac: A New Composite Score With Potential Predictive Value in Prostate Cancer
Source: Front Oncol. 2021 Mar 16;11:644665. doi: 10.3389/fonc.2021.644665 (PMC8009179; doi:10.3389/fonc.2021.644665)

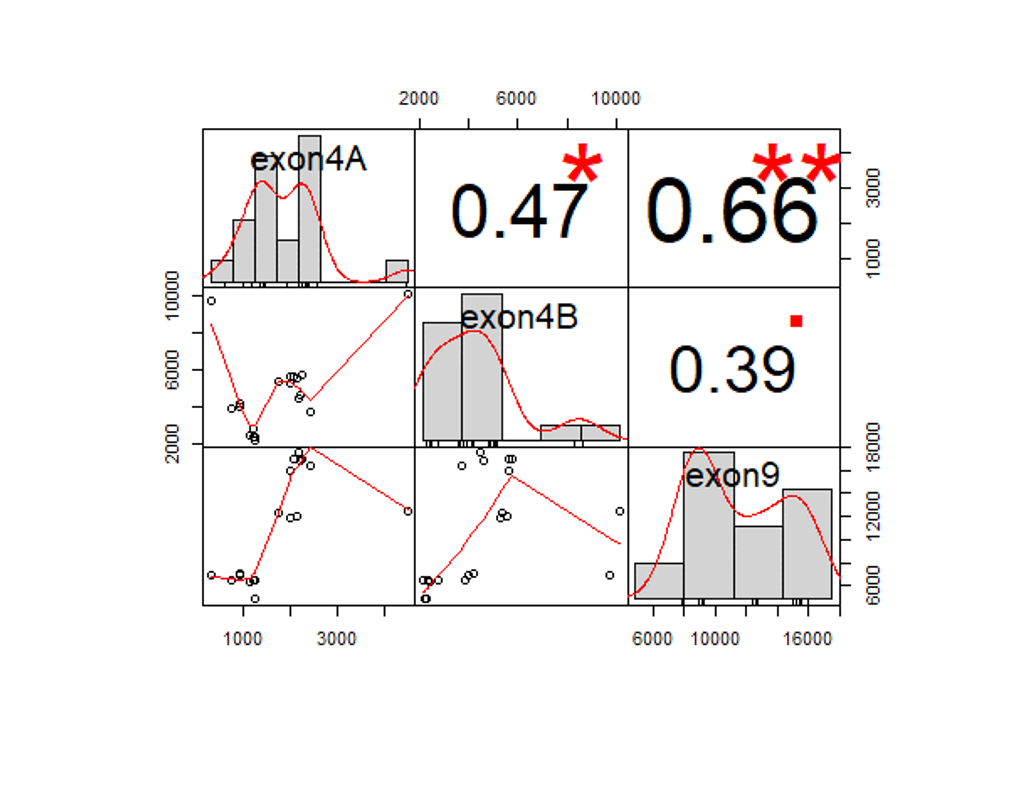

Supplement: Supplementary Figure 1 — Correlation tests between exons. Pearson correlation matrix for PVT1 exons 4A, 4B, and 9. Mapping from p-values to symbols is as follows: ***(0-0.001), **(0.001-0.01), *(0.01-0.05), (0.05-0.1). Histograms show the distribution of the data. [file Image_1.tif]
