# Supplementary material for: Prostac: A New Composite Score With Potential Predictive Value in Prostate Cancer
Source: Front Oncol. 2021 Mar 16;11:644665. doi: 10.3389/fonc.2021.644665 (PMC8009179; doi:10.3389/fonc.2021.644665)
Supplement: Supplementary Table 1 — Measured Copies/µl of exon 4A, 4B, and 9 in copies/µl. [file Table_1.docx]

S1 Table. Measured Copies/µl of exon 4A, 4B, and 9 in copies/µl.

| **Cell Line** | **PVT1 exon 4A** | **PVT1 exon 4B** | **PVT1 exon 9** |
| --- | --- | --- | --- |
| RWPE1_1 | 728 | 3894 | 6403 |
| RWPE1_2 | 920 | 4015 | 6865 |
| RWPE1_3 | 926 | 4181 | 7015 |
| RWPE1_4 | 311 | 9733 | 6856 |
| RWPE1_5 | 1245 | 2166 | 6486 |
| RWPE1_6 | 1241 | 2198 | 4827 |
| RWPE1_7 | 1228 | 2320 | 4859 |
| RWPE1_8 | 1225 | 2778 | 6503 |
| RWPE1_9 | 1214 | 2332 | 6421 |
| RWPE1_10 | 1136 | 2456 | 6396 |
| MDA PCa 2b_1 | 1990 | 5313 | 11911 |
| MDA PCa 2b_2 | 1740 | 5375 | 12237 |
| MDA PCa 2b_3 | 2145 | 5592 | 11973 |
| MDA PCa 2b_4 | 4489 | 10117 | 12509 |
| MDA PCa 2b_5 | 2438 | 3750 | 16436 |
| MDA PCa 2b_6 | 2194 | 4592 | 16790 |
| MDA PCa 2b_7 | 2162 | 4495 | 17524 |
| MDA PCa 2b_8 | 2084 | 5631 | 16924 |
| MDA PCa 2b_9 | 2245 | 5776 | 16911 |
| MDA PCa 2b_10 | 2005 | 5664 | 15997 |
